# Observations on Cataract

**Published:** 1803-02-01

**Authors:** 

**Affiliations:** Member of the Royal College of Surgeons


### *Observations on Cataract; communicated by* Mr. Morley, *Member of the Royal College of Surgeons.*

In the case of Elizabeth Rimmington, which appeared in your Journal for December, Mr. Leeson was disappointed at the failure of the operation which he performed. The eye, after it, having no visible appearance of disease, and the iris contracting or dilating on the admission or exclusion of light, this gentleman supposes the remaining defect of vision to be occasioned by the capsule of the chrystalline lens not being absorbed, as there was no apparent opacity of this membrane, so as to obstruct the rays of light acting upon the retina. I must beg leave to differ from him in that respect from what experience I have had in diseases of this nature; I sometimes have had reason to suppose cataracts were combined with amaurosis; sometimes the latter disease only existed in one eye, although both were attacked by the former. Now, if a person is not very cautious in his examination, he perchance may operate upon the wrong one; and notwithstanding the operation is performed with the greatest judgment, and the lens completely removed, still he will be disappointed, and his ultimate wishes will not be obtained. The neglect which may occasion this misfortune is, not closing the eye-lids of one during the examination of the other; for if both are exposed exposed to the stimulus of light at the same instant, the iris of both will be mutually affected. The above observations are not founded merely upon hypothesis, but upon the following fact. A woman, some little time back, applied to me for the restoration of sight; on examining, I found nearly the whole cornea of one eye to be opake, in the other a complete cataract, and the iris dilating and contracting to the different shades of light; but, when I closed the one with the opake cornea, no such action took place; I immediately concluded amaurosis was the cause, and the motion of the iris was occasioned by the rays of light insinuating themselves obliquely between the opake cornea and tunica albuginea of the other; why this should happen, I leave to the better judgment of others, probably the case of Elizabeth Rimmington still remaining in darkness is gutta serena in the eye which has been operated upon.

*Wellingan, December* 21, 1802.

